# *Omics Notebook*: robust, reproducible and flexible automated multiomics exploratory analysis and reporting

**DOI:** 10.1093/bioadv/vbab024

**Published:** 2021-09-21

**Authors:** Benjamin C Blum, Andrew Emili

**Affiliations:** 1 Center for Network Systems Biology, Boston University, Boston, MA 02118, USA; 2 Boston University School of Medicine, Boston University, Boston, MA 02118, USA; 3 Department of Biology, Boston University, Boston, MA 02215, USA

## Abstract

**Summary:**

Mass spectrometry is an increasingly important tool for the global interrogation of diverse biomolecules. Unfortunately, the complexity of downstream data analysis is a major challenge for the routine use of these data by investigators from broader training backgrounds. Omics Notebook is an open-source framework for exploratory analysis, reporting and integrating multiomic data that are automated, reproducible and customizable. Built-in functions allow the processing of proteomic data from MaxQuant and metabolomic data from XCMS, along with other omics data in standardized input formats as specified in the documentation. In addition, the use of containerization manages R package installation requirements and is tailored for shared high-performance computing or cloud environments.

**Availability and implementation:**

Omics Notebook is implemented in Python and R and is available for download from https://github.com/cnsb-boston/Omics_Notebook with additional documentation under a GNU GPLv3 license.

**Supplementary information:**

Supplementary data are available at *Bioinformatics Advances* online.

## 1 Introduction

There is an increasing computational burden and bottleneck in the high-dimensional interrogation of biological systems created by advancements in engineering and instrumentation, which make global molecular profiling routine (Manzoni *et al.*, 2018). While nucleic acid (DNA and RNA) sequencing-based technologies are still the most common, mass spectrometry (MS)-based profiling of the proteome and metabolome are gaining traction due to the orthogonal information they provide (Perez-Riverol *et al.*, 2019). Integrative analysis of the resulting high-dimensional datasets poses a steep learning curve, however, which motivates the development of automated software tools to unlock functional insights in diverse biological contexts.

Existing tools have succeeded in accelerating research but leave an unaddressed gap, particularly in extracting actionable biological insights from proteomic and multiomic analysis and in shared computing ecosystems used by many core and specialized labs (Haas *et al.*, 2017; Huber *et al.*, 2015). Perseus, a companion to MaxQuant for processing MS-proteomic data, is one widely used tool (Tyanova and Cox, 2018), with a graphical interface and open (though proprietary) framework to facilitate ease of use and expandability, respectively. However, this comes at the expense of limiting automation to accelerate reporting and improve reproducibility. R packages, such as Proteus, MSstats and mixOmics, provide powerful statistical tools for custom analysis but require knowledge of the R-language and are limited in scope, rather than providing an overall framework (Choi *et al.*, 2014; Gierlinski *et al.*, 2018; Rohart *et al.*, 2017). ProVision succeeds in demonstrating the utility of new, guided platforms to accelerate proteomic data analysis and provides a user-friendly and flexible web interface, but the web interface is not optimized for shared high-performance computing (HPC) environments, extension to an entire multiomic workflow, or sharing custom analysis (Gallant *et al.*, 2020). Recent research has demonstrated RStudio projects are a viable medium for sharing data and custom analysis to enable robust information sharing and improve the utility of existing studies (Leprevost *et al.*, 2014; Reznik *et al.*, 2018). The use of containers or workflow managers solves the reproducibility challenges presented by R package dependencies (Boettiger and Eddelbuettel, 2017; Decan *et al.*, 2016). Therefore, a multiomic data analysis tool formatted as an RStudio project and making use of containerization could facilitate code sharing, integrate a wide array of R packages, and be automated to deliver reproducible exploratory analysis and reporting for high volume core labs and research centers.

Here, we introduce Omics Notebook, an open-source laboratory notebook for multiomics data analysis and reporting, which includes multiple expandable modules for reporting and visualizing MS-based data. Omics Notebook is formatted as an RStudio project with code to automate analysis and includes tie-ins to leading bioinformatics R packages. The platform is geared toward analyzing stable isotope (e.g. tandem mass tag)-labeled or label-free protein and peptide/phosphosite data generated from MaxQuant/Andromeda, and other proteomics software/search engines, as well as untargeted metabolomic data from tools such as XCMS, with user-friendly scripts for automating exploratory analysis and reporting on local computers or shared computing resources. We distinguish our software from existing tools (e.g. Perseus and MSstats) by describing the automated analysis, which is invaluable for MS research centers with many high-throughput projects, as well as a thorough exploratory reporting of altered pathways and biological functional modules. Additional omics data, such as transcriptomics, may be analyzed using standardized data formats specified in the documentation. This platform catalyzes biological insights by making use of R markdown to generate reports, images and files for analysis and easy sharing by biologists, which has the potential to accelerate biomedical research.

## 2 Results

**Fig. 1.**
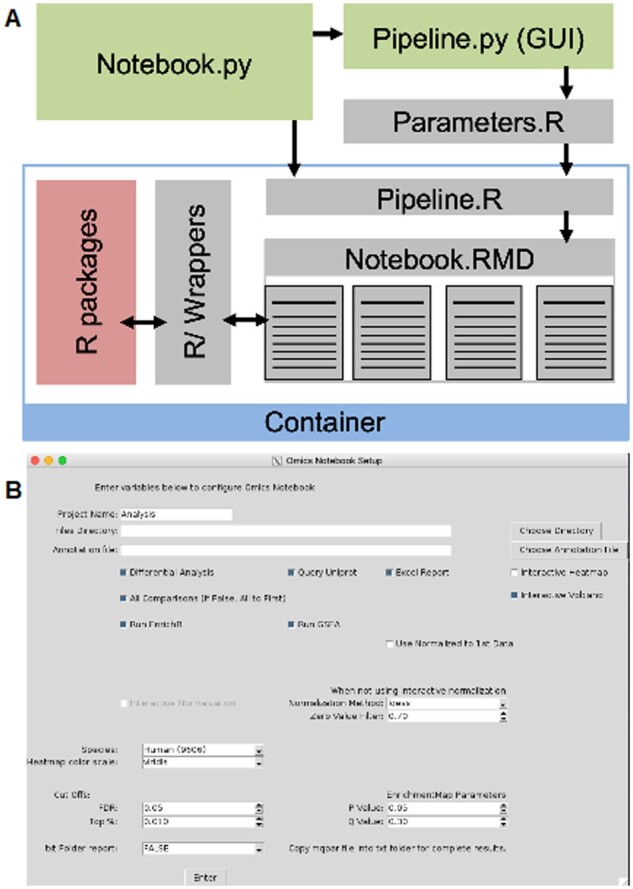
*Omics Notebook* software framework and representative analysis outputs. (**A**) Schematic of major software components. (**B**) Screenshot of GUI parameter input, see Supplementary information for a larger image with annotation

Omics Notebook was created around two major components written in R. First, a series of R markdown documents are used to run the analysis pipeline, which are broken down into major segments (Fig. 1A) and generate custom HTML reports for the analysis. The parent Notebook.Rmd file handles importing data and annotation information, as well as run parameters and formatting. The parent file then calls child Notebook files covering (i) Normalization, Quality Control, and Exploratory Analysis, (ii) Differential Analysis, (iii) Enrichment Analysis and (iv) Integrative Analysis. All run parameters and options are captured in the output R markdown report to facilitate transparency and parameter optimization (e.g. normalization method). Workflow managers (e.g. KNIME or Nextflow) are powerful tools for constructing pipelines but add system dependencies (e.g. Java), which complicate implementation. Therefore, the core of our pipeline is constructed in R itself; however, workflow tools may be useful for integrating Omics Notebook into larger (e.g. raw data processing) pipelines. The second major component is the code in R/, which contains wrapper functions to provide an interface between a common data format in Omics Notebooks and several R packages, which vary substantially in their expected input formats.

Around this core codebase, additional components extend usability by automating input parameters with a graphical user interface (GUI) (Fig. 1B; using Python/tkinter) to provide helpful defaults but allow for optimizing a streamlined set of parameters (see SupplementaryInformation.pdf in the project repository under docs/for larger screenshots with parameter explanations). A Dockerfile is used to create container images to manage the installation compatibility and requirements for a large and diverse set of R packages, each with their own dependencies and requirements. Containerization with Docker and Singularity enable use on cloud and shared HPC computing resources, respectively. A standard annotation file specified which omics data types are present and which files/columns correspond to input sample data. The software will determine which and how many data types are present based on the rows in the annotation file (see Supplementary Annotation file section in the documentation for more information). The software will perform analysis with a minimum of one dataset, and maximum that is limited by the memory constraints of the host environment (and has been tested with up to six datasets of the same experiment). Input data should be in text file formats (either tab or comma delimited) with columns corresponding to samples and rows corresponding to features (e.g. proteins or metabolites). Additional columns represent row annotation and the data matrix should be in a numerical format. The results of transcriptomic or microarray experiments can also be analyzed in Omics Notebook, provided annotation columns follow the same convention described in the documentation and the data are formatted in text file numeric matrices, as described for other data types, or the software is edited to add additional preprocessing steps.

In total, this software framework separates Omics Notebook from existing tools discussed earlier. While other tools, such as Perseus, MSstats and ProVision, offer great feature sets, they are designed to have analysis steps built up manually and separately for each dataset. With Omics Notebook, exploratory, differential and enrichment analysis steps are all automated for rapid reporting to support high-throughput MS research centers. This may not be the best fit for all applications, but can rapidly accelerate bioinformatic analysis when researchers collaborate on many projects that all follow similar designs.

**Fig. 2.**
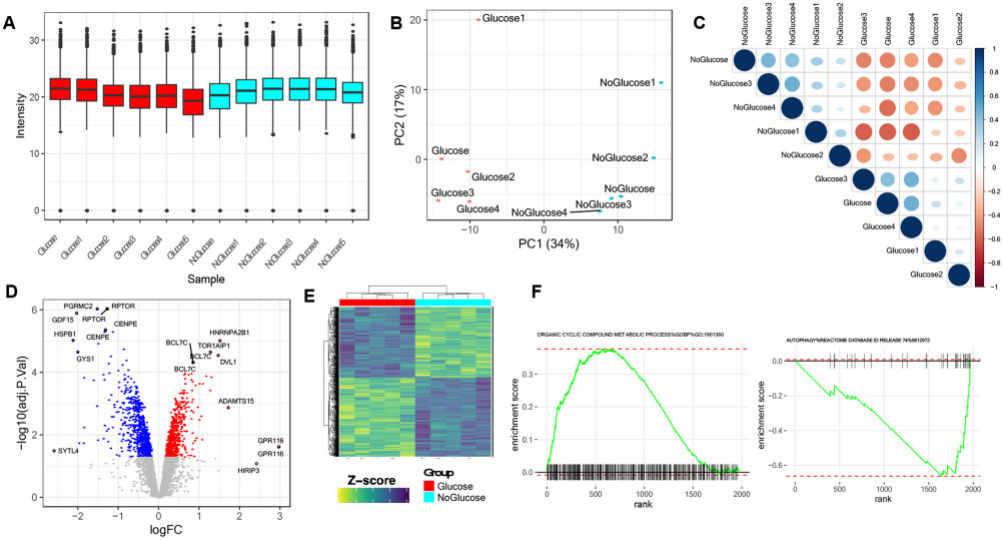
*Omics Notebook* representative analysis outputs. Various output graphs include (**A**) abundance distribution plots, (**B**) dimensionality reduction (e.g. principal component analysis) and (**C**) sample correlation plots. Output from differential analysis includes (**D**) volcano plots and (**E**) heatmaps. Output also includes exploratory enrichment analysis (**F**) with standardized tables and enrichment plots

Critical output from the Omics Notebook pipeline includes visualizations to ensure optimal normalizations and global exploratory analysis (Fig. 2A). Normalized data and additional information are exported in output files and plots, such as principle component analysis (PCA), provide useful global representation (Fig. 2B and C). Differential analysis is powered by the limma R package (Ritchie *et al.*, 2015) and underpins the generation of additional figures, like volcano plots and heatmaps (Fig. 2D and E). While customizable by editing or adding onto the R code, the software comes with many built-in features. For example, users are able to highlight particular features of interest (see documentation), which are reflected in the output plots. Enrichment analysis is performed with several methods to provide optimal in-depth functional exploratory analysis. Enrichr and gene set enrichment analysis (GSEA) (Fig. 2F) are used to provide pathway information for data annotated with Gene symbols (Chen *et al.*, 2013; Korotkevich *et al.*, 2019; Reimand *et al.*, 2019). When multiple datasets with gene symbols are present, enrichment is carried out based on both combined ranked list and statistical data fusion techniques. Kinase-substrate enrichment is automated for phosphosite data (e.g. MaxQuant Phosphosite files) and metabolomic pathway enrichment analysis is performed by MetaboAnalystR (Casado *et al.*, 2013; Chong and Xia, 2018). Integrative analysis occurs across datasets at the pathway level between metabolomic and other data types based on pathway information from metabolic models. The immediate benefit of integrative multiomic analysis is that signals can be aggregated across data types to enable researchers to look for coherent functional patterns.

Additional key exports include a formatted excel file output is generated with critical annotation information to facilitate data sharing with collaborators while avoiding common pitfalls (Ziemann *et al.*, 2016). Additional file outputs with the prefix ‘Network_’ include formatted information to aid in the overlay of experimental data in molecular network analysis tools, such as Cytoscape. Enrichment results are exported in a standardized format that also enables subsequent network analysis of pathways and functional themes or modules (Reimand *et al.*, 2019).

Example data and analysis output are provided in the Omics Notebook project repository (under example/). The example data include protein level data, phosphosite data and two different metabolomic data (acquired with negative and positive ionization, respectively), as reflected in the four rows in the lower section of the annotation file. The processed data are all in tab-delimited (comma delimited also supported) text file formats with matrices consisting of different samples in columns and features in rows. Additional columns in the data are included as row annotations and sample annotation data are imported through the annotation file. The initial stages of analysis, such as data import, formatting and normalization, occur on each dataset separately. Exploratory analysis across datasets, such as correlation, occurs across datasets with common feature identifiers, such as gene symbols in the case of the protein and phosphosite data. Pathway enrichment analysis is carried out first on individual datasets, then combined across datasets with matching feature types, using gene symbols in the protein and phosphosite data, and metabolite features in the metabolomic data. Ultimately, pathway enrichment based on metabolic models is carried out across all available datasets.

Documentation is available in the github repo (under docs/) where there is a thorough readme file, as well as a PDF with supplementary information and example screenshots (https://github.com/cnsb-boston/Omics_Notebook/tree/main/docs). Documentation is also available at readthedocs.io (https://omics-notebook.readthedocs.io). Omics Notebook is tested successfully in an HPC computing environment to provide rapid exploratory analysis and reporting across dozens of diverse projects and input data, as well as to support custom analysis to support multiple high-quality studies already in publication. Additional testing has been performed on local computers running linux, macOS and Windows 10. The software is designed to be installed and customized by researchers with computational experience, however, once implemented, the interface is simple enough to permit use by researchers with more modest bioinformatic expertise.

## 3 Conclusions

Omics Notebook is an open-source data-science reporting framework for MS-centric multiomics data analysis that is designed for ease of use and reproducibility in an automated reporting context, as well as extensive customization to support a wide range of custom data analysis workflows. In addition to routine, automated reporting, Omics Notebook provides multiple options for incorporating analysis-specific parameters, custom options and additional analysis tools available as R packages. The codebase available as an RStudio project facilitates routine code and container sharing to increase reproducibility. In addition, the current code base offers many features that apply high-quality statistical methods and visualizations based on the formatting of data specific to less common multiomics (e.g. MS) studies. Containerization and the standardized reporting allow users to optimize parameters and revisit previous analyses to optimize the insights gained from existing data. This software is designed to be easily customized to suit the needs of specific labs or research centers and to operate in environments where batch processing is common (e.g. cloud or HPC), however, the underlying code could be well-suited to power an interactive Shiny app on a public server, with relatively minor modifications. Omics Notebook accelerates functional analysis of many high-throughput biological experiments, however, a limitation of this and similar approaches is the determination of interpretation or biological significance. While the software succeeds in expediting and alleviating a bottleneck in bioinformatic analysis, researchers must still digest the results and formulate hypotheses about the underlying biological significance, which may require additional project-specific analysis. However, by rapidly extracting experimental signal and reporting on functional patterns within and across datasets, the software enables researchers to spend more time where it will have the greatest impact.

The underlying R scripts and R markdown documents, as well as complete wrapper software, are available at https://github.com/cnsb-boston/Omics_Notebook, along with additional documentation and example data with analysis output.

## Supplementary Material

vbab024_Supplementary_Data
