# Supplementary material for: Omics Notebook: robust, reproducible and flexible automated multiomics exploratory analysis and reporting
Source: Bioinform Adv. 2021 Sep 21;1(1):vbab024. doi: 10.1093/bioadv/vbab024 (PMC9710639; doi:10.1093/bioadv/vbab024)
Supplement: vbab024_Supplementary_Data [file vbab024_supplementary_data.pdf]

Annotation File

The annotation file provides a standard way to read in the experimental data.

The top section has information about what differential analyses to perform, what Group each sample belongs to, and the sample names for each sample. Data set names, "type" and filename should be specified in the bottom left, with no empty rows. Each row in the lower section is a different omics dataset. For a given row, the columns next to SampleName should correspond to the specific samples in the data. Other columns will be kept as annotation information.

In the second sheet of the annotation file, the sample names are repeated. Additional rows can specify additional annotation information. A couple will control additional functionality.

* Batch : if "Batch" is found, the row will be used to try to perform batch correction. The default is to use ComBat. Care should be take not to overfit data.
* ColorsHex : if "ColorsHex" is used, and the row has functional hex colors values corresponding to "Group", these hex colors will be used in figures where possible.
* Group2: if "Group2" is used, this will specify shapes in the PCA plot. More functionality may be added later.
* TimeSeries: if a numeric value, will run limma as for a time series analysis. Will only work with 2 Groups. Code may be edited for other analyses. If there is 1 group, enter the time point as "Group."
* Pairs: if "Pairs" is found, will try to run differential analysis accounting for paired samples.

This list can be extended for custom analysis.

Parts of Annotation File Main Sheet:

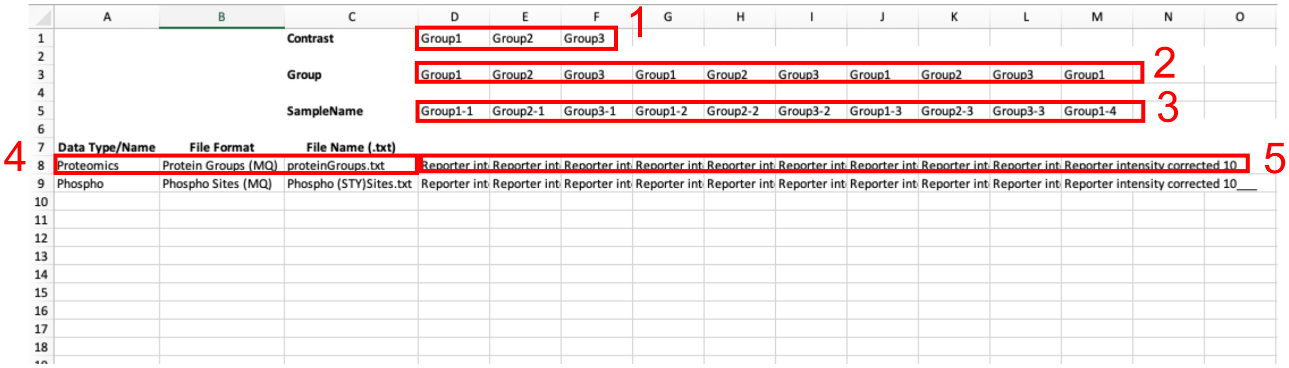

1. This row is the only row that stands alone and doesn't map to columns in rows below. Include one instance of group names in this row if they should be included in the differential analysis. If not running differential analysis, do not leave blank, include at least one group name. The order impacts the directionality of differential analysis. In the example above, the contrasts run will be Group2 – Group1, Group3 – Group1, and Group3 – Group2. If, for example, there is a pool or standard group you don't want to include in the differential analysis, do not include it on this row.
2. This row labels each sample with a group name and corresponds to the samples below. Samples in the same group should have identical names in this row.
3. Sample names provide short, easy to read names for the samples in the data specified below. In many instances, the columns in the input data may have longer or harder to read headers. The sample names here should be short to facilitate used in plots.
4. Information should be provided here about the input data. Data Type/Name is a short name that is easy to read to label the data set in filenames and plots. File format is a drop down list that maps to specific data format inputs so the software will know how to handle formatting. File name specifies the name of the file in the Analysis Directory and should include the file extension (.txt or .csv).

5. Column headers providing the data column for the sample specified above in sample names.

Additional rows (sections 4 and 5) are available for additional multi-omic data analysis.

GUI for Generating Parameters.R

A graphical user interface (GUI) is provided using Python and tkinter to generate a Parameters.R file with correct formatting required to run the analysis. Defaults are provided to accelerate analysis but offer customization of critical options to suit a wide range of data.

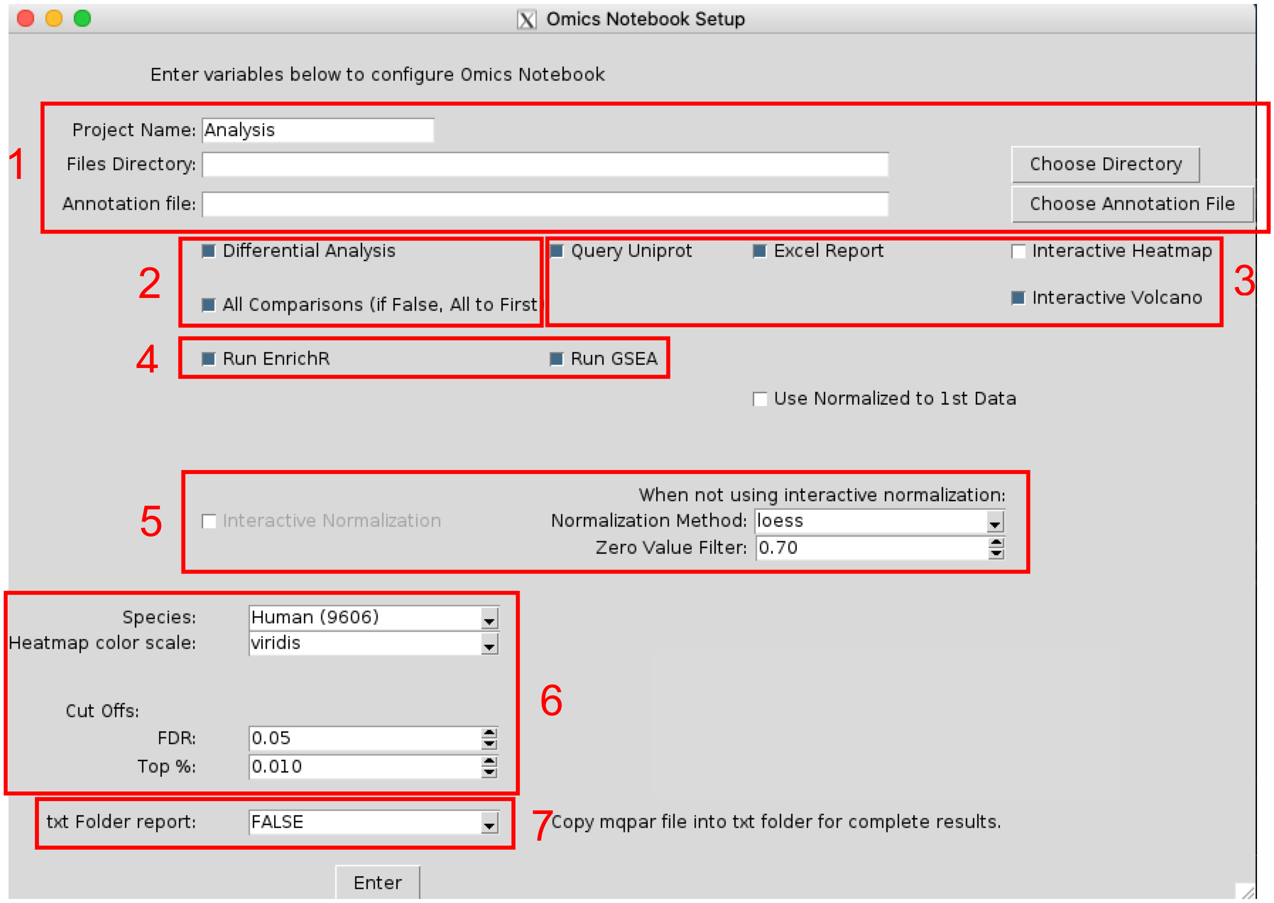

1. Provide a custom name for this analysis, if desired. Output will automatically be labelled with date, so this is useful if running multiple analyses on same day (e.g., with different

parameters). Additionally, provide the Analysis Directory where the data is and output will be saved (requires write permissions) and may be different from Notebook (software) directory. Finally, specify the Annotation file for the analysis.

2. Option to run the differential analysis (checked is True). If there are many conditions/groups, it may make analysis more manageable to run all groups compared to the first condition (i.e., a control group). This should correspond to the first group listed in the contrast row in the annotation file.
3. Options (checked is true) to query uniprot over the internet for selected feature annotation information (works with Uniprot protein id's, like those provided in MaxQuant searches based on Uniprot databases with correct formatting), to generate an excel output file for sharing normalized data and feature level annotation, to generate interactive outputs (heatmaps and volcano).
4. Options to run enrichment analysis based on Enrichr and GSEA.
5. Normalization parameters specifying method, log transformation, and fraction of non-zero values required to retain a feature.
6. Additional analysis parameters to specify species (used for some enrichment analysis), the desired heatmap color scale, and cutoffs for significant value plots.
7. If analyzing MaxQuant data, the entire txt output folder can be provided in the analysis directory, which will be analyzed using the PYXQC R package for quality control information.
